# Autoantibodies against the β_3_-Adrenoceptor Protect from Cardiac Dysfunction in a Rat Model of Pressure Overload

**DOI:** 10.1371/journal.pone.0078207

**Published:** 2013-10-11

**Authors:** Jin Wang, Meixia Li, Xiurui Ma, Kehua Bai, Li Wang, Zi Yan, Tingting Lv, Zhiqing Zhao, Rongrui Zhao, Huirong Liu

**Affiliations:** 1 Department of Physiology, Shanxi Medical University, Taiyuan, Shanxi, P. R. China; 2 State Key Laboratory of Brain and Cognitive Science, Institute of Biophysics, Chinese Academy of Science, Beijing, P. R. China; 3 Shanxi Cardiovascular Diseases Hospital, Taiyuan, Shanxi, P. R. China; 4 School of Basic Medical Sciences, Cardiovascular Research Institute, Capital Medical University, Beijing, P. R. China; 5 Department of Basic Biomedical Sciences, Mercer University School of Medicine, Savannah, Georgia, United States of America; University Hospital of Würzburg, Germany

## Abstract

β_3_-adrenoceptors (β_3_-ARs) mediate a negative inotropic effect in human ventricular cardiomyocytes, which is opposite to that of β_1_- and β_2_-ARs. It has been previously demonstrated that autoantibodies against the β_1/_β_2_-AR exist in the sera of some patients with heart failure (HF) and these autoantibodies display agonist-like effects. Our aim in this study was to observe whether autoantibodies against the β_3_-AR (β_3_-AR Abs) exist in the sera of patients with HF and to assess the effects of β_3_-AR Abs on rat model of pressure overload cardiomyopthy. In the present study, the level of β_3_-AR Abs in the sera of HF patients was screened by ELISA. β_3_-AR Abs from HF patients were administrated to male adult rats with abdominal aortic banding (AAB), and the cardiac function was measured by echocardiographic examination and hemodynamic studies. The biological effects of this autoantibody on cardiomyocytes were evaluated using a motion-edge detection system, intracellular calcium transient assay, and patch clamp techniques. Compared to healthy subjects, the frequency of occurrence and titer of β_3_-AR Abs in the sera of HF patients were greatly increased, and β_3_-AR Abs could prevent LV dilation and improve the cardiac function of rats with AAB. β_3_-AR Abs exhibited negative chronotropic and inotropic effects and were accompanied by a decreased intracellular Ca^2+^ transient and membrane L-type Ca^2+^ current in cardiomyocytes. Our results demonstrated the existence of β_3_-AR Abs in the sera of patients with HF and found that this autoantibody could alleviate the cardiac dysfunction induced by pressure-overload in AAB rats.

## Introduction

Heart failure (HF) is a life-threatening clinical condition in which the heart cannot pump enough blood to the rest of the body. The clinical symptoms of HF include water-sodium retention, decreased perfusion of peripheral tissues and organs, which are the common final phase of many cardiovascular diseases [1]. Despite the improvement of medical therapy, the clinical outcome is extremely poor [2]. The main problem is its heterogeneous in etiology and pathogenesis. Among them, dysregulation of the β-adrenergic system has been considered to play a critical role in the development of cardiac dysfunction associated with HF [3-5].

In recent years, autoantibodies against the β_1_- and β_2_- adrenoceptor (AR) have been detected in the sera of patients with chronic HF [6-9]. These autoantibodies are specifically directed against the second extracellular loop of human β_1_- and β_2_-ARs and display agonist-like activities [6,9-13]. Furthermore, immunization by peptides corresponding to the target sequences of the anti-receptor autoantibodies induced morphological and functional changes in the rat or rabbit heart similar to those observed in patients with HF [14-18]. These studies suggest that autoanbibodies against the G-protein-coupled receptors have important pathophysiologic role in the occurrence and development process of HF [19].

β_3_-AR is a newly-identified cardiac adrenoceptor that belongs to the superfamily of G protein-coupled-receptors [20]. However, β_3_-AR differs from classical β_1_- and β_2_-AR by its opposite roles in the regulation of cardiac functions. The β_3_-AR has been found in the human ventricular myocardium where they produce a negative inotropic effect that was mediated through Gi proteins [21]. Moreover, in contrast to the downregulation of β_1_- and β_2_-AR during the development of HF [22], β_3_-AR proteins were markedly increased (2- to 3- fold) in failing compared with non-failing hearts, and a similar increase was also observed for Gi proteins that coupled β_3_-AR to their negative inotropic effect [23-26]. Furthermore, Rasmussen [27] and Niu [28] et al. have demonstrated that β_3_-AR agonists could improve cardiac function of HF patients and a lack of β_3_-AR could exacerbate LV dilation and dysfunction [29]. These results suggest that activation of β_3_-AR may also play an important role in the modulation of cardiac function in HF. As β_3_-AR also belongs to the G protein-coupled receptor family [20], we speculated that it may have similar immunological characteristics with β_1_-AR/β_2_-AR, and that autoantibodies against the β_3_-AR (β_3_-AR Abs) may also exist in the sera of HF patients. If this assumption is valid, what is the effect of β_3_-AR Abs on cardiac function?

Therefore, the purposes of the present study were 1) to determine whether HF patients could produce β_3_-AR Abs, 2) to investigate whether β_3_-AR Abs could affect the cardiac function in rats with abdominal aortic banding, and 3) to study the biological activities of these autoantibodies on cardiomyocytes in an attempt to explore its possible mechanisms.

## Methods

The research protocol was approved by the Institutional Committee for the Protection of Human Subjects of Shanxi Medical University Hospital. All patients were informed about the purpose and protocol of the study, and written consent was obtained. The study adheres to the principles of the Declaration of Helsinki and Title 45, US Code of Federal Regulations, Part 46, Protection of Human Subjects, revised 13 November 2001, effective from 13 December 2001. All experimental procedures and protocols were approved by the Ethics Committee and Animal Welfare Committee of Shanxi Medical University.

### Patient Characteristics

Sera from 76 patients with HF were collected from the First and Second Hospitals of Shanxi Medical University, Taiyuan, China. The diagnosis of HF was based on the patients’ clinical history, physical examination, echocardiography, left ventriculography, electrocardiogram, chest radiography, and coronary angiography according to WHO/ISFC [30]. For purposes of comparison, sera from 100 healthy subjects were obtained from normal healthy volunteers at the First Hospital of Shanxi Medical University. The protocol was approved by the Research Committee of Shanxi Medical University. The sera were collected and stored at −20°C for subsequent detection of β_3_-AR Abs. The characteristics of patients with HF and healthy subjects are summarized in Table 1. 

**Table 1:** Characteristics of patients with HF and healthy subjects.

**Characteristics**	**HF(n=76)**	**Control(n=100)**
*Age(y)*	*58.8±8.6*	*52.8±10.6*
Gender(male/female)	48/28	66/34
Heart rate (beats/min)	76±11	72±13
SBP(mmHg)	117±18	119±15
DBP(mmHg)	73±13	75±13
Functional class (NYHA)*		
I	9	100
II	32	0
III	21	0
IV	14	0
Ejection fraction (%)	37.2±6.7^*^	70.5±8.6
Plasma BNP (pg/ml)	328±45^*^	32.4±16.7
**Medications**		
ACE inhibitors / ARBs	63	0
β-Blockers	58	0
Diuretics	32	0

HF: Heart Failure; NYHA: New York Heart Association. ^*^ *P* < 0.05 *vs.* healthy subjects.

### Peptides

A peptide corresponding to the sequence (residues 176-202) of the second extracellular loop of the human β_3_-AR [31] with a cysteine as carboxy terminus (QWWRVGADAEAQRCHSNPRCCAFASNMC) was synthesized by Meilian Bioengineering Company, Xian, China. 

### ELISA

50 μl of peptide (5 μg/ml) in a 0.1 mol/L Na_2_CO_3_ solution (pH 11.0) were coated on a 96-well microplate overnight at 4°C. The wells were then saturated with PBS supplemented with 5% bovine serum and 0.1% Tween 20 (PMT). 50 μl of sera dilutions from 1:10 to 1:160 in PMT were allowed to react with the peptide for 1 hour at 37°C. After washing three times with PBS, 0.05 ml of biotinylated rabbit anti-human IgG antibody (1:1000 dilution in PMT) was allowed to react for 1 hour at 37°C. After three washings, the bound biotinylated antibody was detected by incubation of the plates for 1 hour with streptavidin-peroxidase (1 μg/ml) solution in PMT. This was followed by three washings in PBS and addition of substrate (2.5 mmol/L H_2_O_2_, 2 mmol/L ABTS, Sigma Immunochemicals). Optical densities (O.D.) were read after 30 min at 405 nm in a microplate reader. The positivity of the sera to the peptide was defined as P/N≥2.1 (P/N=specimen O.D.- blank O.D./negative control O.D.-blank control O.D.). The antibody titer was determined by the continuous double dilution of the samples from 1:10 and expressed as the maximum dilution when P/N≥2.1 [32]. 

### Purification of IgG

Based on a seropositive response in enzyme immunoassay to peptide 176-202 of the β_3_-AR, immunoglobulin fractions (IgGs) from these positive sera were prepared using a MabTrap Kit (Amersham) by following the manufacturer’s instructions. The concentration of purified IgGs was determined by using a Coomassie blue detection kit (Jiancheng Bioengineering Company, Nanjing, China). The specificity of the purified IgGs was determined by ELISA.

### Immunofluorescence Staining

Cultured H9c2 cells were washed with PBS (pH 7.4) and fixed with 4% paraformaldehyde for 20min at 37°C. Cells were blocked with 5% bovine serum albumin (BSA) in PBS (w/v) for 1 hour at 37°C. Then, the cells were incubated overnight at 4°C with β_3_-AR antibody (Abcam, UK) and the IgG fractions (25 μg/ml) from β_3_-AR Abs positive HF patients, respectively. Following three PBS washes, cells were incubated with FITC-labeled secondary antibodies for 1 hour in the dark at 37°C. After being rinsed with PBS, cover slips with mounting medium containing DAPI stain nuclei were coated. Negative controls were performed by omitting primary antibodies. Fluorescence images were acquired and analyzed using an Olympus FV 1000 Confocal microscope.

### Abdominal aortic banding surgery

Abdominal aortic banding (AAB) was induced by standard methods [33]. Briefly, Wistar rats (10 weeks old, weighing 200-220g) were chosen and anaesthetized with 10% chloral hydrate solution (30 mg/kg i.p.) and with aseptic surgical procedures. For the banding model, we opened the abdomen and separated the abdominal aorta, placed a 0.7 mm needle adjacent to the isolated aortic segment, tightly banded the aorta with an adjacent needle, and then drew out the needle. The sham control group underwent the same procedures without constriction of the aorta. 

Group-I: control, sham-operated; Group-II: Untreated abdominal aortic banding (AAB) rats; Group-III: AAB rats treated with β_3_-AR Abs via tail vein injection, 2 μg/g; Group-IV: AAB rats treated with negative IgGs via tail vein injection, 2 μg/g. IgGs were administered once every 10 days and the total period was 8 weeks.

### Echocardiographic examination

In vivo cardiac function and geometry were assessed by transthoracic echocardiography (VIVID 7 dimension system, General Electric-Vingmed Ultrasound). The rats were anesthetized with methoxyflurane by inhalation. Left ventricular (LV) end-systolic and end-diastolic cross-sectional diameter (LVESD, LVEDD), and the mean of septal and posterior wall thicknesses were recorded from M-mode images. LV fractional shortening (FS) and LV ejection fraction (EF) were determined as previously described [34] .

### Hemodynamic Studies

Hemodynamic parameters were measured by cardiac catheterization [35]. Left ventricular systolic pressure (LVSP), left ventricular end diastolic pressure (LVEDP), and maximal rate of rise and decline of ventricular pressure (±dp/dt_max_) were recorded and analyzed.

### Culture of neonatal beating cardiomyocytes

The hearts were removed aseptically from 1- to 2-day-old Wistar rats and the isolated cardiomyocytes were cultured as previously described [36]. Briefly, single cells were dissociated from the minced ventricles with a 0.25% solution of trypsin and were cultured at 37°C for 4 days as monolayers. On the day of the experiment, the medium was replaced and the cells were incubated at 37°C for 2 hours. Thereafter, the beating frequency of the spontaneously beating cardiomyocytes was measured on a heated stage of an inverted microscope at 37°C. The number of beats of a selected isolated myocardial cell or a cluster of synchronously contracting cells in each of 10 fields was counted for 15 sec each time. The changes of beating frequency were measured 5 min after the addition of the tested agents. The basal beating rate was 92.67±10.86 beats per minute. The data represented observations on 10 to 30 cells or cell clusters of synchronously beating cardiomyocytes in three different cultures.

### Cell isolation procedure

Single ventricular cardiomyocytes were enzymatically isolated from the rat hearts as described previously [37]. Briefly, the rats were decapitated and the hearts were rapidly excised and mounted onto a Langendorff perfusion apparatus and were immediately perfused with Ca^2+^-free Tyrode solution (in mmol/L: 143 NaCl, 5.4 KCl, 0.5 MgCl_2_, 0.3 NaH_2_PO_4_, 5.0 HEPES, 5.0 glucose, pH 7.4) equilibrated with O_2_ until spontaneous contractions ceased. Subsequently the heart was perfused with Ca^2+^-free Tyrode solution containing 0.4 g/L collagenase II (270U/mL) and 0.7 g/L bovine serum albumin (BSA) for about 20 min until it became soft and then followed by 5 min perfusion with Ca^2+^-free Tyrode solution to remove the enzyme. Ventricles were separated and minced in Krebs solution (in mmol/L: 70 L-glutamic acid, 25 KCl, 20 Taurine, 10 KH_2_PO_4_, 3.0MgCl_2_, 0.5 EGTA, 10 HEPES, 10 glucose, pH 7.4) supplemented with 2% BSA before being filtered through a nylon mesh (200 mesh). The viable cells were subsequently separated by sedimentation for 10 min, twice. The ventricular cardiomyocytes were then re-suspended in the Krebs solution supplemented with 2% BSA, and Ca^2+^ was slowly added to the cell suspension until it reached a final concentration of 1.8 mmol/L. Typically, about 70-80% rod-shaped cardiomyocytes were obtained.

### Cell shortening/re-lengthening assay

 The contraction and intracellular Ca^2+^ transient of ventricular cardiomyocytes were assessed by a video-based motion edge detection system (IonOptix, USA) [38]. Cells were placed in a chamber mounted on the stage of an inverted microscope (Olympus) and superfused (1 ml/min, 25°C) with Tyrode solution. The cells were field-stimulated at a frequency of 0.5 Hz at a 5 ms duration using a pair of platinum electrodes placed on the opposite sides of the chamber. The cardiomyocyte being studied was displayed on the computer monitor imaged through a 40× objective using an IonOptix Myocam camera. Criteria for choosing cardiomyocytes for the experiment include: i) a rod shape, ii) clearly defined sarcomeric striations, iii) steadily contracted in response to electrical stimulation and without spontaneous contractions, and iv) a stable steady-state contraction amplitude for at least 5 min before drug administration. Cell shortening and re-lengthening were assessed by the following indices: peak twitch amplitude (PTA, % cell length), time to 90% peak shortening (TPS), time to 90% re-lengthening (TR_90_), and velocities of shortening (-dL/dt) and re-lengthening (+dL/dt).

### Intracellular fluorescence measurement

Cardiomyocytes were loaded with fura-2/AM (0.5 µmol/L) for 30 min in the dark at room temperature. The fluorescence measurement was then recorded by a dual-excitation fluorescence photomultiplier system (IonOptix). Cells were exposed to light emitted by a 75 W lamp and passed through either a 360- or a 380-nm filter, while being stimulated to contract at 0.5 Hz. Fluorescence emission was detected at 510 nm by a photomultiplier tube after first illuminating the cells at 360 nm then at 380 nm for the duration of the recording protocol. The 360 nm excitation scan was repeated at the end of the protocol and qualitative changes in intracellular Ca^2+^ concentration were inferred from the ratio of the fluorescence intensity at the two wavelengths [39].

### Electrophysiological measurements

Single ventricular cardiomyocytes were obtained using an enzymatic dissociation procedure similar to that described previously [40]. The whole-cell clamp technique was used for recording the membrane currents. An aliquot of the cell suspension was placed in a recording chamber on the stage of an inverted microscope (XDP-1, Shanghai) and perfused constantly at a rate of 1-2 ml/min with Tyrode’s solution. The electrodes were pulled in two stages by the two-step progress patch pipette puller (Narishige, Japan). For the measurement of I_Ca-L_, the pipette solution (in mmol/L) contained egtazic acid (EGTA) 10.0, KCl 140.0, Na_2_-ATP 2.0, HEPES 5.0, 4-aminopyridine (4-AP, Sigma) 5.0, MgCl_2_ 1.0 (PH 7.3 adjusted with KOH). The glass pipette has a resistance of 2-4 megaohms after filling with the pipette solution. A whole-cell ‘giga ohm seal’ recording was used as described by Hamill et al. [41]. The pipette was connected to a patch-clamp amplifier (Axopatch-200A, Axon Instrument, USA). Computer program pClamp 5.51 (Axon Instrument, USA) was used to produce voltage clamping signals. Analysis was carried out by program pClampfit 8.0. 

### Reagents

BRL37344 (a selective β_3_-AR agonist), Bupranolol, a nonselective β-AR antagonist, Nadolol (a β_1_- and β_2_- AR antagonist) was provided by Dr. Zhi-Qing Zhao (Mercer University School of Medicine, USA) and Dr. Che-Ping Cheng (Bowman Gray Medical College, USA). 

### Statistical Analysis

All data are expressed as means ± SD. Results were analyzed by Independent Samples t Test, chi square test or one-way ANOVA where appropriate using SPSS 11.5 statistics software. Probabilities of 0.05 or less were considered statistically significant.

## Results

### Prevalence and β_3_-AR Abs titers in sera from HF and healthy subjects

**Figure 1:**
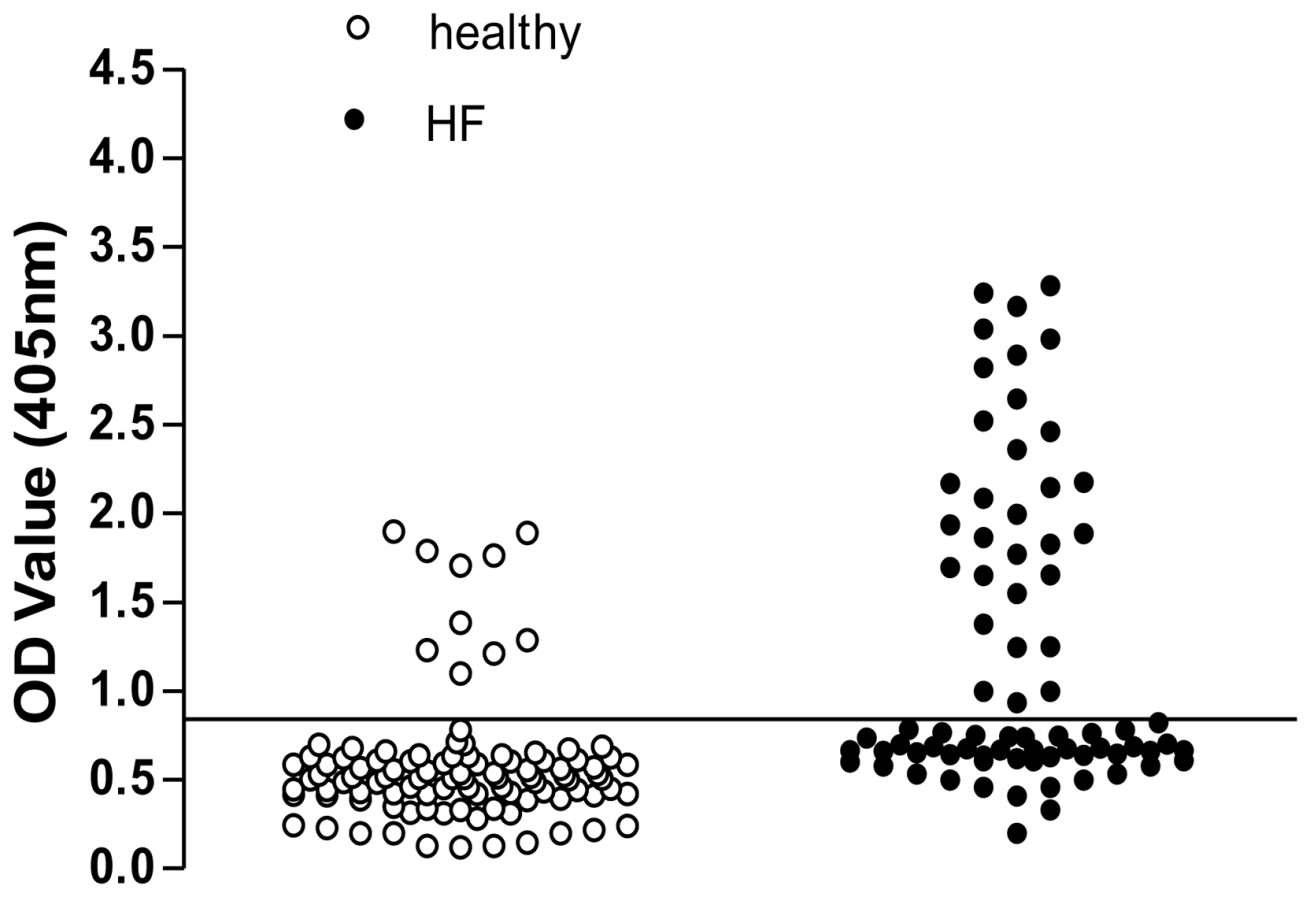
Detection of β_3_-AR Abs in HF patients and healthy subjects. Scatter diagram demonstrating optical density values (dilution of 1:10) for detecting β_3_-AR Abs in each serum sample from 76 HF patients and 100 healthy subjects.

A scatter diagram of optical density (OD) values was displayed in Figure 1. In the sera of 100 healthy subjects, 11 (11%) were positive for β_3_-AR Abs. However, 31 (40.8%) of 76 HF patient sera was positive for β_3_-AR Abs, a significantly greater prevalence than in healthy subjects (*P* < 0.001). The geometric mean of positive β_3_-AR Abs titers in HF patients was also significantly greater than that of healthy subjects (1:75.9 ± 1.89 in HF patients *vs.* 1:13.8 ± 1.72 in control group, *P* < 0.001).

### β_3_-AR Abs could bind to β_3_-ARs on the surface of H9c2 cells

**Figure 2:**
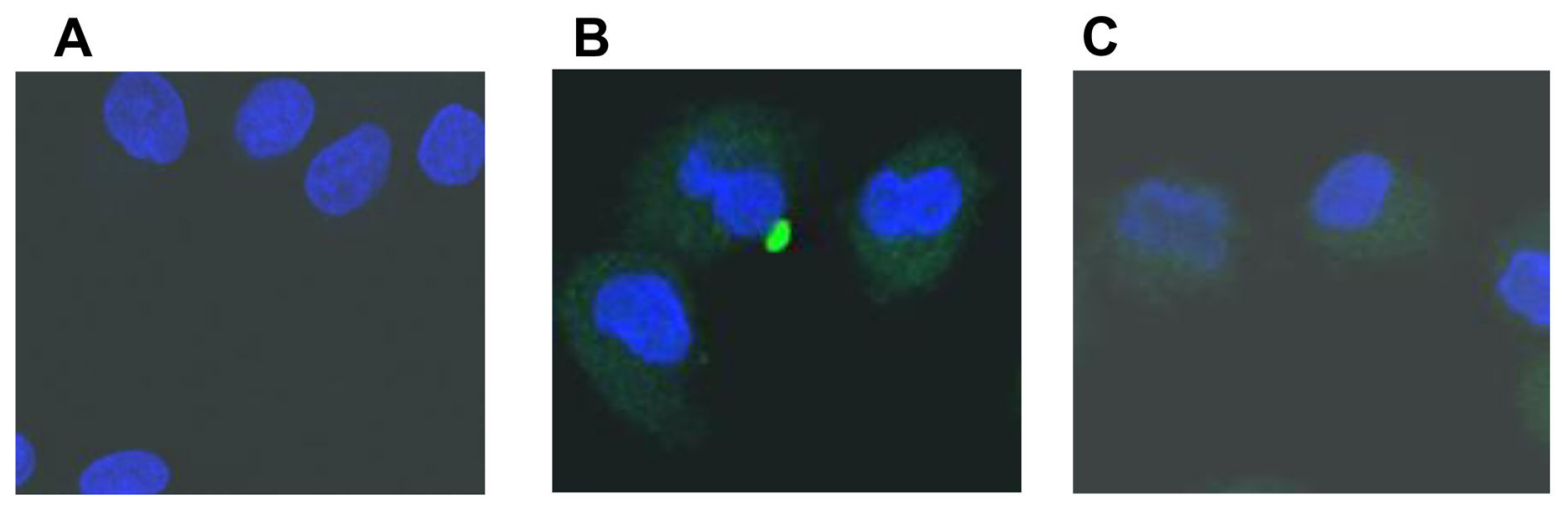
β_3_-AR Abs from HF patients bound to β_3_-AR on the surface of H9c2 cells. A. The negative control. Nuclei were labeled with DAPI (blue). B. The positive control. Nuclei (blue), β_3_-AR was identified with anti-β_3_-AR antibody (Abcam, UK) (green). C. The experiment group. Nuclei (blue), β_3_-AR was identified with β_3_-AR Abs from HF patients (green).

Immunofluorescence staining was used to determine whether the IgG fraction isolated from the β_3_-AR Abs positive sera of HF patients could bind to β_3_-ARs of rats. The result showed that β_3_-AR Abs from HF patients was mainly bound to the cell membrane, and the binding pattern of β_3_-AR Abs with β_3_-AR was virtually identical to commercial β_3_-AR specific antibodies (Figure 2A, B, C). 

### β_3_-AR Abs could improve cardiac function in rats undergoing abdominal aortic banding (AAB) surgery

**Figure 3:**
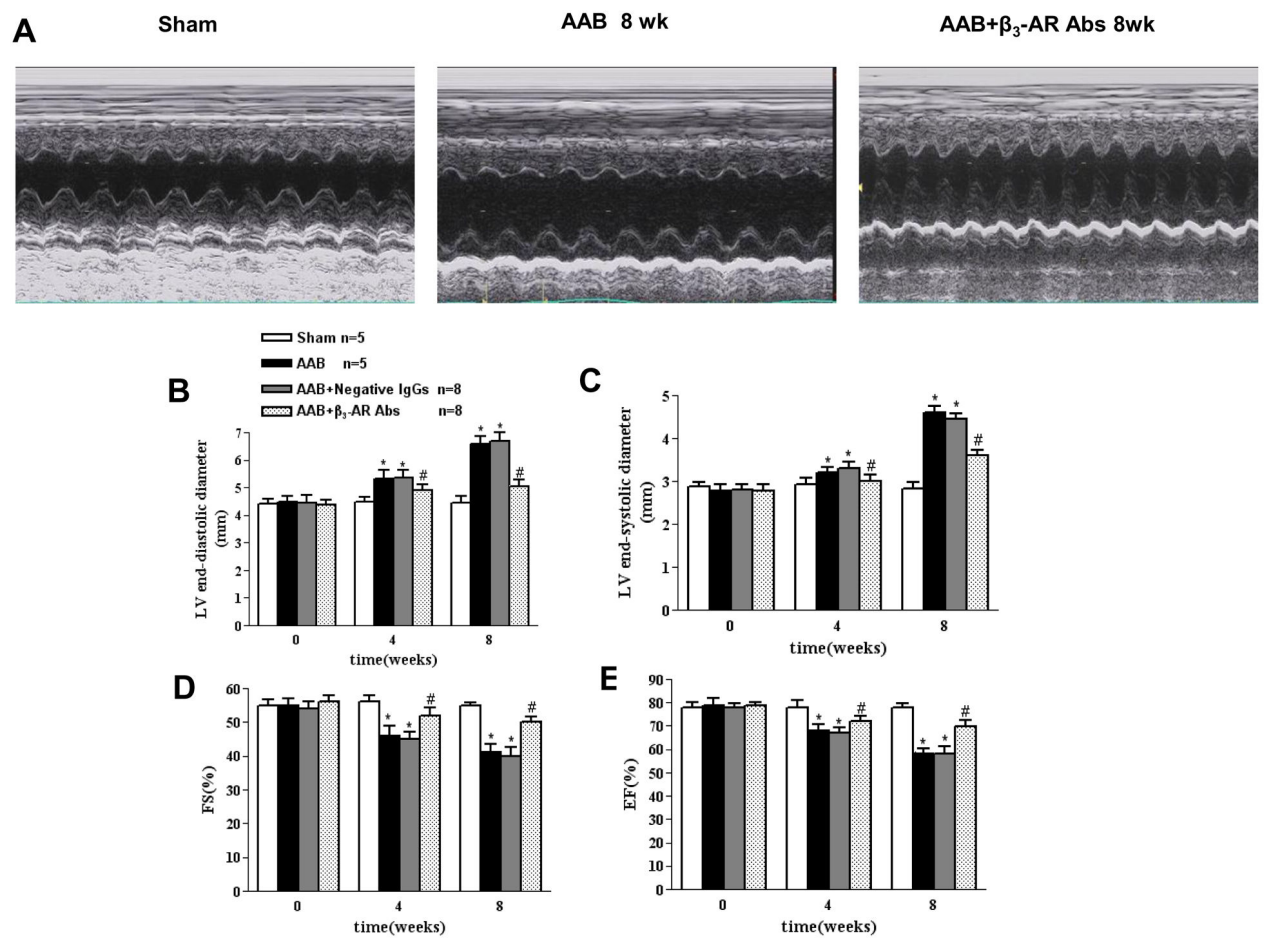
Effects of β_3_-AR Abs on LV dilation and systolic function in AAB rats by echocardiography. A. M-mode echocardiography images from Sham-operated (Sham), 8wk post-AAB, and 8wk post-AAB treated with β_3_-AR Abs. B. C. β_3_-AR Abs prevented LV chamber dilation induced by AAB. D. E β_3_-AR Abs ameliorated LV systolic dysfunction induced by AAB. ^*^ *P*<0.05 *vs*. Sham-operated group, ^#^ *P*<0.05 *vs*. AAB group. Sham n=5, AAB n=5, AAB+β_3_-AR Abs n=8, AAB+Negative IgGs n=8. FS: fractional shortening, EF: ejection fraction.

Echocardiography data showed that LV end-diastolic dimensions (LVEDD) and end-systolic dimensions (LVESD) progressively increased in AAB rats at 4 weeks (LVEDD 5.32±0.27 mm *vs.* 4.28±0.19 mm, LVESD 3.62±0.23 mm *vs.* 2.66±0.21 mm, *P*<0.05 *vs.* sham-operated group) and increased further at 8 weeks (Figure 3A, 3B and 3C). The fractional shortening (FS%) and ejection fraction (EF%) significantly decreased at 4 weeks and decreased further at 8 weeks (Figure 3D, 3E) compared with the sham-operated rats. In contrast, AAB rats treated with β_3_-AR Abs prevented LV dilation (*P*<0.05 for both LVEDD and LVESD), and ameliorated the decrease in FS% and EF% compared with the AAB group at 4 and 8 weeks. However, the AAB rats treated with negative IgGs did not show significant effect on cardiac function. (Figure 3B, 3C, 3D, 3E)

**Figure 4:**
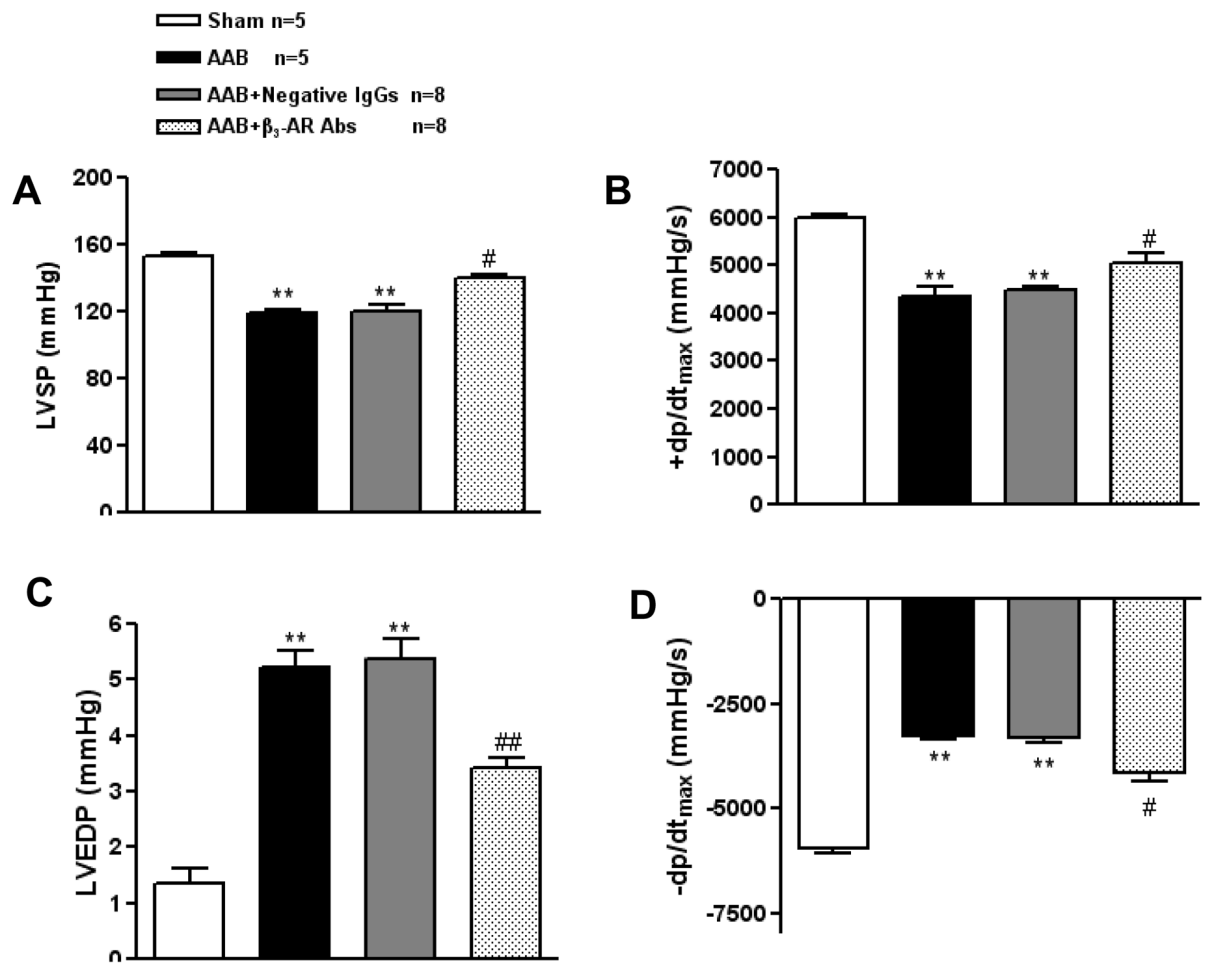
Effects of β_3_-AR Abs on cardiac function in AAB rats by LV hemodynamic analysis. The change of LVSP (A), +dp/dt_max_ (B), LVEDP (C), and -dp/dt_max_ (D) in AAB rats treated with β_3_-AR Abs. ^*^ *P*<0.05, ^**^ *P*<0.01 *vs*. Sham-operated group, ^#^ *P*<0.05, ^##^ *P*<0.01 *vs*. AAB group. Sham n=5, AAB n=5, AAB+β_3_-AR Abs n=8, AAB+Negative IgGs n=8.

In addition, cardiac function was evaluated by LV hemodynamic analysis at 8 weeks after AAB. The AAB rats showed a lower left ventricular systolic pressure (LVSP), and a higher left ventricular end diastolic pressure (LVEDP) compared to the sham-operated rats, and the AAB rats also showed a depressed left ventricular ±dp/dt_max_. Conversely, the AAB rats treated with β_3_-AR Abs showed an increased LVSP and a reduced LVEDP accompanied with a higher ±dp/dt_max_ compared with AAB rats, and AAB rats treated with negative IgGs showed no obvious changes. (Figure 4A, 4B, 4C, 4D)

Taken together, these data indicate that β_3_-AR Abs could improve cardiac function in rats with abdominal aortic banding.

### β_3_-AR Abs had a negative chronotropic and negative inotropic effects on rat cardiomyocytes

**Figure 5:**
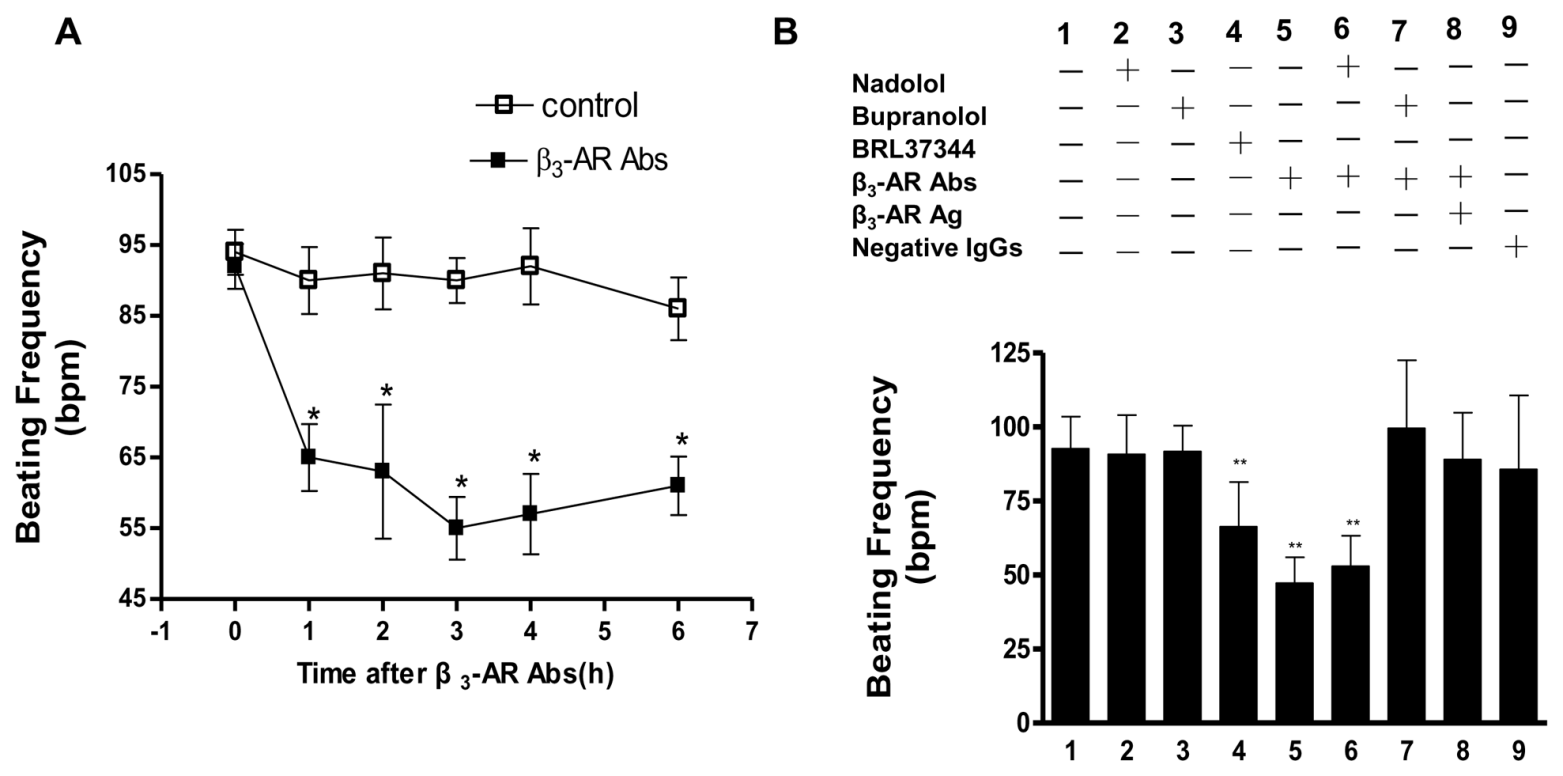
Negative chronotropic effects of β_3_-AR Abs in cultured neonatal rat cardiomyocytes. A. Negative chronotropic effects of β_3_-AR Abs (0.1 µmol/L) upon beating frequency of neonatal rat cardiomyocytes at different time points. ^*^ *P* < 0.05, ^**^P < 0.01. B. Negative chronotropic actions of β_3_-AR Abs (0.1 µmol/L) on beating frequency of neonatal rat cardiomyocytes after one hour exposure. The effects of nadolol, bupranolol, and β_3_-AR antigen peptide on the actions of β_3_-AR Abs are shown. ^**^ *P* < 0.01 *vs*. control. (n=10).

To evaluate the potential role played by β_3_-AR Abs in HF pathogenesis and associated cardiac dysfunction, we examined the chronotropic effects of β_3_-AR Abs on the spontaneous beating frequency of neonatal rat cardiomyocytes. As shown in Figure 5A, purified IgGs (0.1 µmol/L) from HF patient sera positive for β_3_-AR Abs markedly decreased cardiomyocyte beating frequency from 93.56±5.47/min to 64.32±8.13/min after 1 hour of exposure, suggesting a negative chronotropic effect of β_3_-AR Abs. The negative chronotropic effects of β_3_-AR Abs reached statistical significance within 1 hour and remained unabated during measurements for up to 6 hours, suggesting a cellular lack of desensitization to β_3_-AR Abs’ chronotropic effects. As summarized in Figure 5B, seronegative IgGs had no effect upon cardiomyocyte beating frequency. However, β_3_-AR Abs exerted a negative chronotropic effect comparable to that of β_3_-AR agonist BRL37344 (0.1 µmol/L). Moreover, the negative chronotropic effect of β_3_-AR Abs was abolished by bupranolol, a nonselective β-AR antagonist, but not by nadolol, a selective β_1_- and β_2_-AR antagonist. Neither nadolol nor bupranolol had any effects upon cardiomyocyte beating frequency. These results provided direct evidence that the negative chronotropic effect was mediated by β_3_-AR, and not β_1_- or β_2_-AR. The specificity of β_3_-AR Abs’ chronotropic effects was also verified by pre-incubation with its corresponding antigen peptide; the negative chronotropic effects of β_3_-AR Abs were completely eliminated by the β_3_-AR antigen peptide.

**Figure 6:**
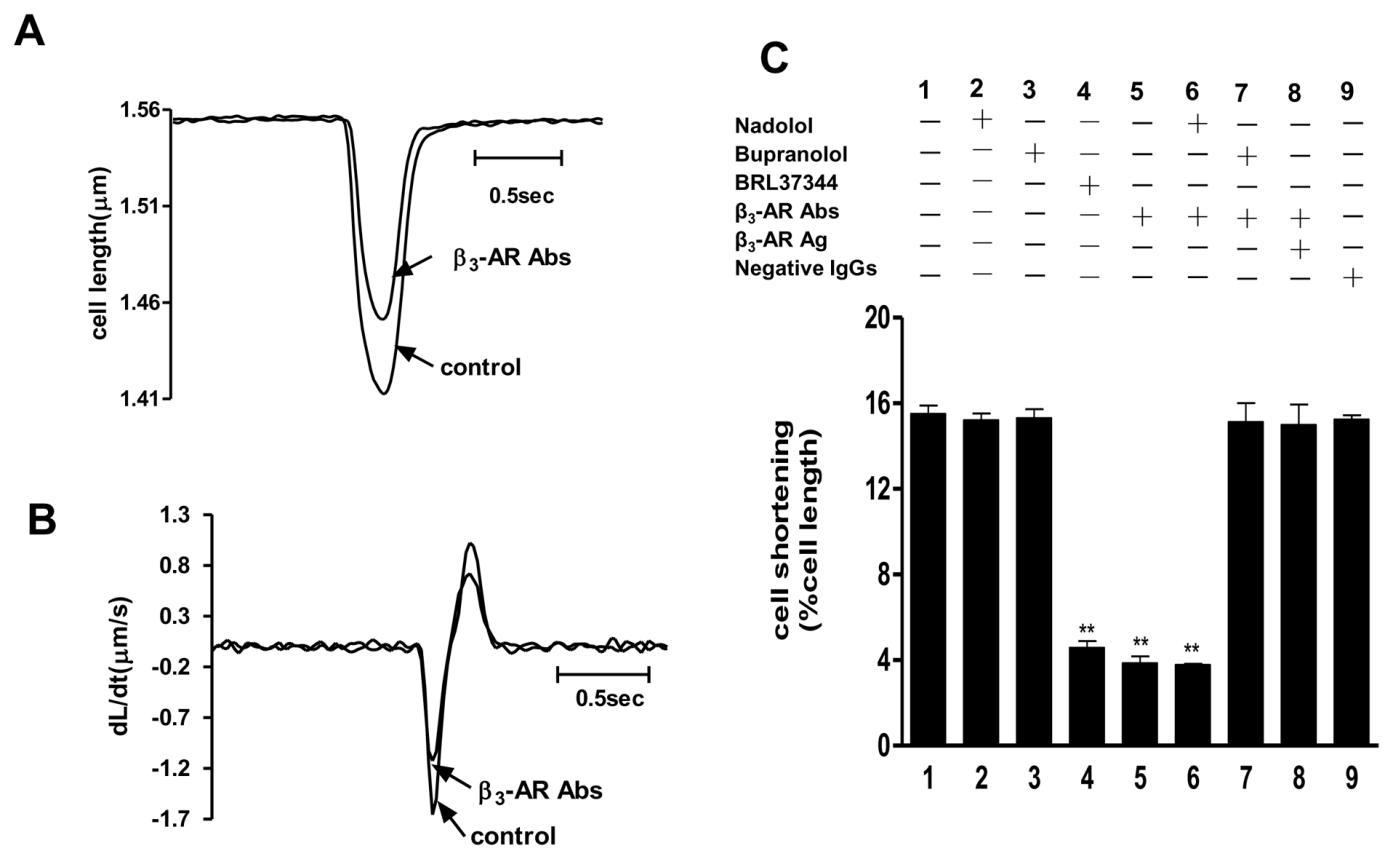
Negative inotropic effect of β_3_-AR Abs on isolated adult rat cardiomyocytes. A and B. Representative profiles of cell length and dL/dt. C. Negative inotropic agonist-like activities of β_3_-AR Abs (0.1 µmol/L, n=10) and the effects of nadolol, bupranolol, and β_3_-AR antigen upon β_3_-AR Abs-induced activities in isolated adult rat cardiomyocytes. (^**^ *P* < 0.01 *vs*. control, n=10).

We concomitantly investigated the inotropic effects of β_3_-AR Abs on isolated adult rat cardiomyocytes. Representative contraction profiles were shown in Figures 6A and 6B, demonstrating the effects of β_3_-AR Abs upon amplitude and velocity of shortening/re-lengthening in isolated rat cardiomyocytes. As summarized in Figure 6C, purified IgGs (0.1 µmol/L) from β_3_-AR Abs positive HF sera manifested agonist-like effects upon cardiomyocyte contraction similar to β_3_-AR agonist BRL37344 (0.1 μmol/L), as evidenced by a decrease in PTA (peak twitch amplitude, % cell length). Again, the nonselective β_3_-AR antagonist bupranalol successfully blocked the inotropic effect of purified IgGs from β_3_-AR Abs positive HF sera, but nadolol, a selective antagonist of β_1_- and β_2_-AR, did not, suggesting the mediation of observed IgG inotropic effects through β_3_-ARs rather than β_1_- or β_2_-ARs. Neither nadolol nor bupranolol had any effects upon cell length. Furthermore, similar to the chronotropic effects experiment, the inotropic effects of purified IgGs from β_3_-AR Abs positive HF patients were completely abolished after pre-incubation with its corresponding β_3_-AR antigen peptide. This result strongly suggests that the negative inotropic effects were induced by the purified IgGs from β_3_-AR Abs positive HF patients and were mediated by β_3_-AR Abs rather than other IgGs present in the patient sera.

**Figure 7:**
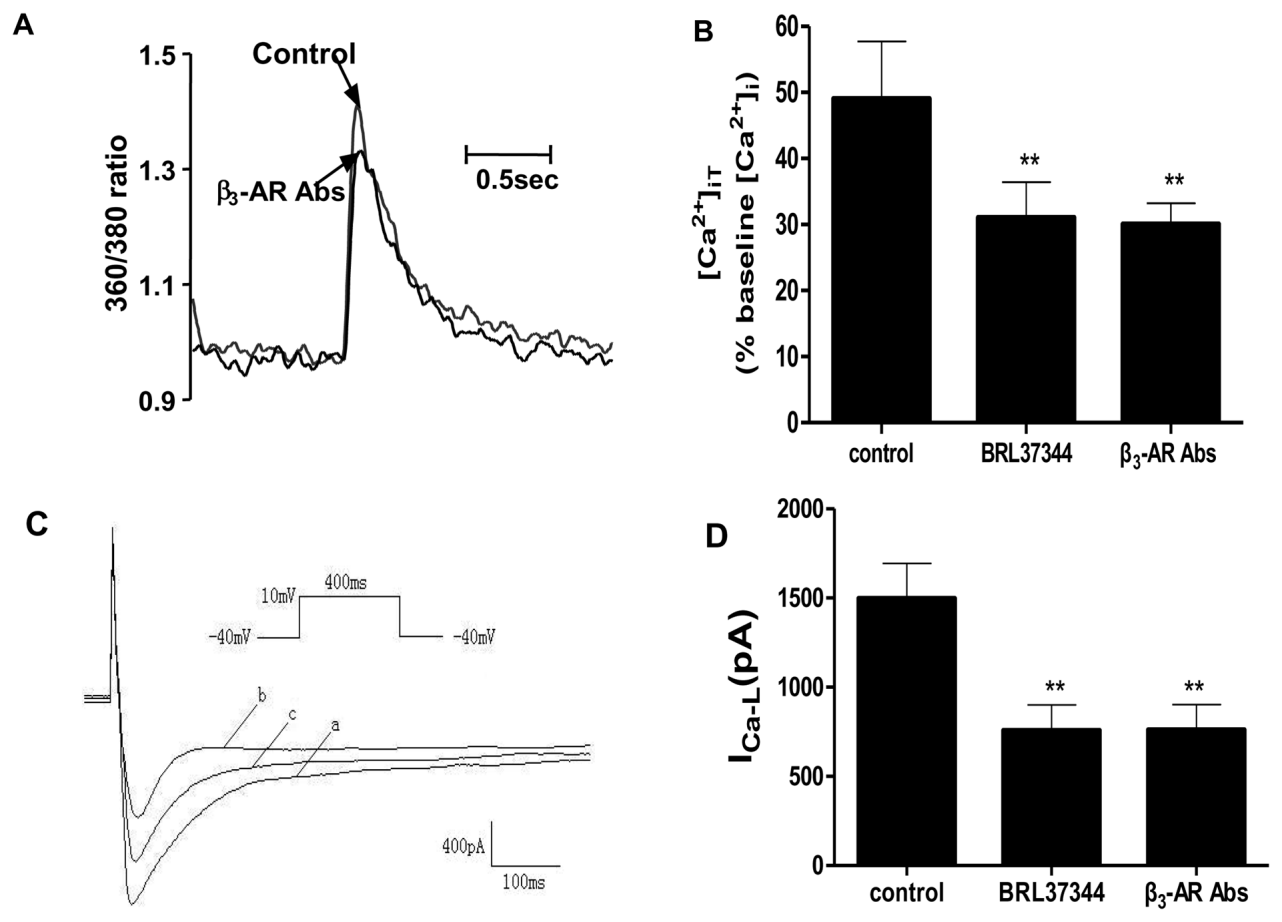
Effects of β_3_-AR Abs on intracellular calcium transient ([Ca^2+^]_iT_) and I_Ca-L_ in isolated adult rat cardiomyocytes. A. Representative [Ca^2+^]_iT_ profile in isolated rat cardiomyocytes demonstrating decreased [Ca^2+^]_iT_ induced by β_3_-AR Abs (0.1 μmol/L). B. Summary data (n=10, ^**^ *P* < 0.01 *vs*. control.) C. Representative I_Ca-L_ profile in isolated rat ventricular cardiomyocytes demonstrating inhibitory effects of β_3_-AR Abs (0.1 µmol/L) on I_Ca-L_. a. control; b. β_3_-AR Abs; c. after β_3_-AR Abs wash out. D. Group mean data demonstrating effects of β_3_-AR Abs (0.1 µmol/L) and BRL37344 (0.1 µmol/L) on I_Ca-L_ in isolated rat ventricular cardiomyocytes. ^**^ *P* < 0.01 *vs*. control. n=5.

To clarify the mechanisms underlying the negative inotropic effects of β_3_-AR Abs, we investigated changes in intracellular calcium transient ([Ca^2+^]_iT_) and L-type Ca^2+^ current (I_Ca-L_) of cardiomyocytes induced by β_3_-AR Abs. A representative [Ca^2+^]_iT_ profile was shown in Figure 7A, demonstrating the effects of β_3_-AR Abs upon cardiomyocytes [Ca^2+^]_iT_. Figure 7B showed decreasing effects of β_3_-AR Abs on [Ca^2+^]_iT_ in isolated rat cardiomyocytes. Purified IgGs (0.1 μmol/L) from β_3_-AR Abs positive HF sera decreased the peak systolic [Ca^2+^]_iT_ from control value of 48.54 ± 12.41% to 30.26 ± 3.34% (*P* < 0.01, n=10) in isolated rat cardiomyocytes. This effect was comparable with that induced by β_3_-AR agonist BRL37344 (0.1 μmol/L, which decreased peak systolic [Ca^2+^]_iT_ from control value of 48.54 ± 12.41% to 32.97 ± 5.81%, *P* < 0.01, n=10). Figure 7C showed an inhibitory effect of β_3_-AR Abs (0.1 µmol/L) on ventricular membrane L-type Ca^2+^ current (I_Ca-L_). As shown in Figure 7D, it was evident that β_3_-AR Abs markedly decreased the peak inflow I_Ca-L_ from control value of 1467 ± 223 to 626 ± 138 pA (*P* < 0.01, n=5). This effect was comparable to that observed with β_3_-AR agonist BRL37344 (0.1 µmol/L), which decreased peak inward I_Ca-L_ from control value of 1467 ± 223 to 574 ± 129 (*P* < 0.01, n=5).

## Discussion

The novel findings in this study were that 1) the occurrence frequency and the OD value of β_3_-AR Abs in patients with HF were much higher than that of healthy subjects, and 2) β_3_-AR Abs could ameliorate cardiac dysfunction in AAB rats, and its negative inotropic and chronotropic effects and inhibition of L-type calcium channels may be responsible for the protective effects.

In this study, the peptide with a sequence corresponding to the second extracellular loop of human β_3_-AR was used as the antigen to screen for β_3_-AR Abs in the sera from patients with HF and healthy control subjects. The reason for choosing the second extracellular loop of the receptor is that this loop has been shown to be highly antigenic, immunogenic and important for receptor function in many G-protein-coupled receptors [6,9,42]. Furthermore, this loop contains the T and B cell epitopes necessary for induction of an immune response [10,43].

Our results showed that 40.8% of patients with HF were β_3_-AR Abs positive. Moreover, the occurrence frequency and the OD value of β_3_-AR Abs in patients with HF were much higher than that of healthy subjects, which were similar to those for anti-β_1_- and β_2_-adrenoceptors or muscarinic M_2_-receptor autoantibodies reported previously by Chiale [9], Liu [42] and Fu [44]. Although autoantibody-producing B-cell clones exist in healthy organisms, they are generally suppressed or activated to a limited extent in normal conditions and are thus insufficient to cause damage or a disease state. Therefore, the presence of autoantibodies at a lower titer does not necessarily reflect a pathological state [42,45]. The higher frequency of occurrence of β_3_-AR Abs in HF patients suggested that this novel autoantibody may play a significant role in the pathogenesis of HF.

In most cases, HF is accompanied by cardiac hypertrophy which is an appropriate adaptive response to maintain adequate function in the presence of chronic pathological stress [46,47]. Although this myocardial enlargement is initially beneficial [48], prolonged hypertrophy may accelerate cardiac dysfunction and HF [49,50]. Consequently, it is necessary to determine whether β_3_-AR Abs could mitigate or accelerate the transition from compensatory cardiac hypertrophy to HF. And a common cause of cardiac hypertrophy is chronic pressure overload due to hypertension or aortic stenosis [51].

Therefore, in the present study, the pressure overload rat model was set up through abdominal aortic banding (AAB), and β_3_-AR Abs purified from HF patients were passively administered to AAB rats to observe whether these autoantibodies could affect the cardiac function. In fact, numerous studies [32,52,53] adopted human-derived antibodies in animal models to examine the biological characteristics of these antibodies. Furthermore, the immunofluorescence technique was used to confirm that β_3_-AR Abs purified from HF patients could recognize β_3_-ARs expressed on rat cardiomyocytes.

In the present study, we observed that administering the β_3_-AR Abs to AAB rats for 4 and 8 weeks could prevent LV chamber dilation and ameliorate cardiac dysfunction, showing the cardioprotective role of β_3_-AR Abs in AAB rats. In order to explore the underlying mechanisms responsible for its cardioprotective effects, we investigated the biological activities of β_3_-AR Abs on cardiomyocyte. Compared with the complex factors affecting the cardiac functions in vivo, the use of the isolated cells was easily manipulated and suitable for the study of direct functional effects of autoantibodies on cardiomyocytes. Our results confirmed the agonist-like activities of these β_3_-AR Abs as evidenced by their negative chronotropic effects in cultured cardiomyocytes without desensitization and negative inotropic effects with decreasing [Ca^2+^]_iT_ and I_Ca-L_ in isolated cardiomyocytes. And the inhibitory effects of β_3_-AR Abs on I_Ca-L_ and [Ca^2+^]_iT_ may be the molecular mechanisms that are responsible for its negative inotropic effects. In addition, our current experimental results demonstrated that the agonist-like effects of β_3_-AR Abs were mediated by β_3_-AR because the chronotropic and inotropic effects of β_3_-AR Abs were completely blocked by bupranolol, a nonselective β-AR antagonist and by β_3_-AR antigen peptide, but not by nadolol, a selective β_1_- and β_2_-AR antagonist. These results suggested that the cardioprotective role of β_3_-AR Abs in pressure-overload hypertrophy may be attributable to its negative chronotropic and negative and inhibition of L-type calcium channels which could reduce myocardial oxygen consumption and intracellular calcium overlod, hence may ameliorate the pressure overload-induced pathological remodeling and cardiac dysfunction.

Previous reports and the present study show that both autoantibodies against the β_1_-AR (β_1_-AR Abs) and β_3_-AR (β_3_-AR Abs) exist in the sera of patients with HF and it is of significance to compare the role of β_1_-AR Abs and β_3_-AR Abs in the development of HF. In recent years, a large number of investigations have demonstrated the involvement of β_1_-AR Abs in the pathogenesis of HF [6-8]. These autoantibodies display a stimulatory agonist-like activity on the target receptors without desensitization [11,12]. Hence, β_1_-AR Abs would permanently overstimulate the β_1_-ARs which result in a large amount of energy consumption and finally lead to HF. In contrast to β_1_-AR, stimulation of β_3_-AR could induce a negative inotropic effect [21]. Moreover, the β_3_-AR is markedly up-regulated in failing heart [23-26] which may reflect a compensatory mechanism to protect the heart from overstimulation of β_1_-AR. Thus the β_3_-AR Abs may serve as a “brake” to protect the heart from catecholamine overstimulation by means of its stimulatory agonist-like activities on β_3_-ARs. In the current study, we confirmed that β_3_-AR Abs from patients with HF exerted a cardioprotective role in the rat failing heart induced by pressure overload. We observed that administering the β_3_-AR Abs to rats with AAB could prevent LV dilation and improve the cardiac function of rats. Our findings are partly supported by several recent reports that β_3_-AR agonists had cardioprotective effects in pressure overload hypertrophy and HF [27,28] and lacking of β_3_-AR showed exacerbated pathological remodeling and impaired cardiac function [29].

In conclusion, we have shown that β_3_-AR Abs had substantial cardioprotective effects in pressure overload hypertrophy, which may alleviate the development of HF. These findings provide new insights concerning the significance of β_3_-AR Abs in the pathogenesis of HF. 

## Limitation

Our work leaves some unanswered questions for future study. Although we observed that β_3_-AR Abs could improve heart function, the mechanisms require further research. 
